# Expression Profile of Developmentally Important Genes in preand peri-Implantation Goat Embryos Produced *In Vitro*

**DOI:** 10.22074/ijfs.2016.4659

**Published:** 2016-09-05

**Authors:** Pouria HosseinNia, Mehdi Hajian, Mojtaba Tahmoorespur, Sayyed Morteza Hosseini, Somayyeh Ostadhosseini, Mohammad Reza Nasiri, Mohammad Hossein Nasr-Esfahani

**Affiliations:** 1 Department of Animal Science, Faculty of Agriculture, Ferdowsi University of Mashhad, Mashhad, Iran; 2 Department of Reproductive Biotechnology, Reproductive Biomedicine Research Center, Royan Institute for Biotechnology, ACECR, Isfahan, Iran

**Keywords:** Goat, Developmental Stage, Gene Expression, Preimplantation

## Abstract

**Background:**

Little is understood about the regulation of gene expression during early goat embryo development. This study investigated the expression profile of 19 genes, known to be critical for early embryo development in mouse and human, at five different stages of goat *in vitro* embryo development (oocyte, 8-16 cell, morula, day-7 blastocyst, and day 14 blastocyst).

**Materials and Methods:**

In this experimental study, stage-specific profiling using real time-quantitative polymerase chain reaction (RT-qPCR) revealed robust and dynamic patterns of stage-specific gene activity that fall into four major clusters depending on their respective mRNA profiles.

**Results:**

The gradual pattern of reduction in the maternally stored transcripts without renewal thereafter (cluster-1: *Lifr1, Bmpr1, Alk4, Id3, Ctnnb, Akt, Oct4, Rex1, Erk1, Smad1* and *5*) implies that their protein products are essential during early cleavages when the goat embryo is silent and reliant to the maternal legacy of mRNA. The potential importance of transcription augment at day-3 (cluster-2: *Fzd, c-Myc, Cdc25a, Sox2*) or day- 14 (cluster-3: *Fgfr4, Nanog*) suggests that they are nascent embryonic mRNAs which intimately involved in the overriding of MET or regulation of blastocyst formation, respectively. The observation of two expression peaks at both day-3 and day-14 (cluster-4: *Gata4, Cdx2*) would imply their potential importance during these two critical stages of preand periimplantation development.

**Conclusion:**

Evolutionary comparison revealed that the selected subset of genes has been rewired in goat and human/goat similarity is greater than the mouse/goat or bovine/goat similarities. The developed profiles provide a resource for comprehensive understanding of goat preimplantation development and pluripotent stem cell engineering as well.

## Introduction

Mammalian preimplantation embryonic development encompasses the period from fertilization to implantation. During this period, the embryonic stages and critical developmental events assessed are transition from germinal vesicle stage (GV) to metaphase-II (MII) oocyte, maternal-to-embryonic transition (MET), and the first lineage differentiation to the inner cell mass (ICM) and trophectoderm (TE) during blastocyst formation (1). Notably, implantation in ungulates, unlike human and mice, occurs with a delay of around 7 days. During this “peri-implantation” period, the rapid development of TE dramatically alters the blastocyst morphology from a sphere to a day 14 hatched blastocyst (2).

An improved understanding of gene activity that regulates preimplantation development is crucially important for assisted reproduction techniques and for derivation of embryonic stem cells (3). This goal has been largely achieved in mouse and human (4, 5). For example, it has been shown that embryonic developmental program is regulated by intricate cooperation of several important genes in the context of cell-signaling pathways. It was initially presumed that the developmental genes regulating early embryonic events are conserved across all mammalian species. Researchers attempted to extrapolate mice and human knowledge databases to the embryonic development of other species as well. However, further comparative studies revealed that species-specific differences exist between gene regulatory networks regulating embryo development in mammals (3, 5-7), which will provide a roadmap for differentiating definitive species-specific differences.

The goat is a valuable livestock animal with promising importance in agriculture, biomedicine and transgenesis (8, 9). However, the molecular basis of goat early embryonic development is poorly understood. Yan et al. (10) for the first time demonstrated that the expression of Oct4 and Nanog proteins were not restricted to the ICM of goat blastocysts. To date, four studies have reported derivation of goat “putative” embryonic stem cells (ESCs) from embryos produced either *in vivo* or *in vitro* (11-14). However, chimera production and germ line transmission of ESC have yet remained to be established in the goat (15).

An improved understanding of expression pro- files of developmentally important genes in pre- and peri-implantation goat embryos would improve current attempts to establish ESC in this valuable farm species. Therefore, this study for the time investigated the expression profile of 19 genes, known to be critical for early embryo development in mouse and human, at five different stages of goat *in vitro* embryo development (oocyte, 8-16 cell, morula, day-7 and 14 blastocysts). 

## Materials and Methods

In this experimental study , unless otherwise stated, all chemicals and media were obtained from Sigma Chemical Co. (St. Louis, MO, USA) and Gibco (Grand Island, NY, USA), respectively.

### Selection of genes set

Nineteen candidate genes for the investigation were selected from the human and mouse data bases if i. They were only present in ESCs and either in the oocyte or blastocyst and ii. Their gene ontology applications indicated a critical role in transcription regulation, pluripotency and differentiation. This gene set included *Lifr1, Bmpr1, Alk4, Id3, Ctnnb, Akt, Oct4, Rex1, Smad1, 5, Fzd, c-Myc, Cdc25a, Sox2, Fgfr4, Nanog, Erk1, Gata4,* and *Cdx2*. Since sequence data of some genes was not available in the goat, we designed specific primers based on ortholog conserved regions in other studies. The registered cDNA for *Erk1, Alk4, Bmpr1, Fgfr4* and *Lifr1* were deposited into NCBI database under accession numbers KC687077, KF039752, KF039753, KF039754, and KF356183, respectively). Sequences and characteristics of successful polymerase chain reaction (PCR) primers can be found in Table 1. 

#### *In vitro* production of goat embryos 

The procedure used for *in vitro* production of goat embryos was as described previously (16). In brief, cumulus-oocyte complexes (COCs) were obtained from abattoir-derived goat ovaries. COCs were cultured in maturation medium comprised of tissue culture medium-199 (TCM199) supplemented with 10% fetal calf serum (FCS), 2.5 mM sodium pyruvate, 100 IU/mL penicillin, 100 µg/mL streptomycin, 10 µg/mL follicle-stimulating hormone (FSH), 10 µg/mL luteinizing hormone (LH), 1 µg/mL estradiol-17β, and 0.1 mM cysteamine under mineral oil for 20-22 hours at 39ºC, 6% CO_2_, and maximum humidity. Matured oocytes were used for *in vitro* fertilization (IVF) and presumptive zygotes were cultured in groups of six in 20 µl droplets of modified formulation of synthetic oviductal fluid (mSOF) at 39ºC, 6% CO_2_, 5% O_2_, and maximum humidity (16, 17). 

For real time-quantitative PCR (RT-qPCR) experiments, oocytes and embryos at five different stages of goat *in vitro* embryo development (MII- oocyte, 8-16 cell, morula, expanded blastocyst, and day 14 blastocyst) were used. MII oocytes were collected at 20-22 hours post maturation. The 8-16-cell embryos, expanded blastocysts and day 14 blastocysts were collected during different days post embryo culture (days 3, 7 and 14.post embryo culture). Therefore, variation effect was removed from samples. After through washing in phosphate buffer saline (PBS), oocytes and embryos in pools of 60 (oocyte), 35-40 (D3), and 20 (D7) were collected in 500 µL microtubes containing 75 µl RLT buffer, frozen and stored at -70ºC until RNA extraction. 

#### Derivation of *in vitro* D14 embryos

**Fig.1:**
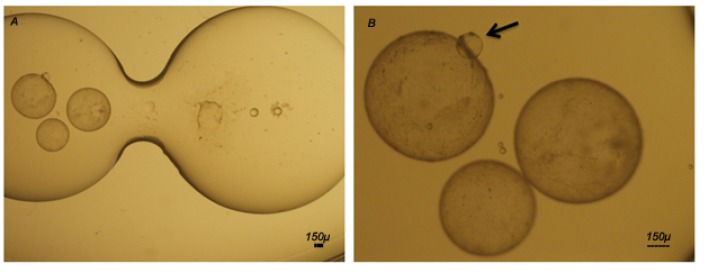
Extended *in vitro* culture system for goat embryos. A. The modified system for culture of expanded goat embryos in medium conditioned by feeder cells and B. Comparison between D7 expanded blastocyst (arrow) with D14 blastocysts developed in the modified culture system.

For extended *in vitro* culture of goat day-7 blastocysts until day-14, we prepared a co-culture system using a feeder layer of caprine fetal fibroblasts (CFF) as described by Behboodi et al. (12). Accordingly, CFF cell-line was prepared using fetal tissues of three 40-day goat fetuses. Single-cell suspension was prepared by mincing fetal tissues and culturing them in Dulbecco’s modified eagle medium (DMEM) supplemented with 10% FCS, 0.25% amphotericin-B, 1% penicillin-streptomycin, 1% gentamycin in 25 cm^2^ culture flasks and cultured at 37ºC, 6% CO_2_ Confluent monolayer was appeared from day 4 of culture onwards. The monolayer was trypsinized and passaged for proliferation of CFF cell-line. Each round of cell proliferation took around 3-4 days. The CFF cell-line at passages 2-to-4 was treated with mitomycin (10 mg/mL) for 2 hours. Treated cells were seeded at 1×10^5^cells/mL in drops of 100 µl DMEM which was placed in close proximity to a feeder-free 100µl droplet of DMEM supplemented with 10% FCS, 1% L-glutamine, 1% non-essential amino acid, and 0.1% β-mercaptoethanol under mineral oil. Five to six D7-blastocysts were transferred to each 100 µl droplet of feeder-free DMEM. Then, the DMEM drops containing blastocysts were gently connected to their adjacent DMEM containing CFF monolayer using a mouse pipette. This culture system provided beneficial effects of feeder layer for extended *in vitro* embryo culture while preventing attachment the day 14 blastocysts to the feeder layer (Fig .1). The culture medium was refreshed every other day until D14 of embryo development. Then, groups of 7-10 well-developed D14 embryos were pooled for RNA extraction as described above. A range of 50-65% of the developed blastocysts could progress to day 14. 

#### RNA extraction and real time-quantitative polymerase chaine reaction 

The procedure for RT-qPCR was as described previously (18). In brief, total RNA of oocytes and embryos was extracted suing RNeasy Micro kit (Qiagen, Mississauga, ON, Canada) followed by treatment with DNase I (Ambion, Streetsville, ON, Canada) according to the manufacturer’s protocol. The RNA quality and quantity was determined using WPA Biowave spectrophotometer (Cambridge, United Kingdom). For reverse transcription, 10 µl of total RNA was used in a final volume of 20 µl reaction containing 1 µl of Random Hexamer, 4 μl RT buffer (10 x), 2 µl of dNTP, 1 μl of RNase inhibitor (20 IU), and 1μl of reverse transcriptase (Fermentas, Glen Burnie, Ontario, Canada). Reverse transcription was carried out at 25°C for 10 minutes, 42°C for 1 hour and 70°C for 10 minutes. 

The selection of appropriate reference gene is of crucial importance in the accuracy and fidelity of the data of RT-qPCR results. Accordingly, we searched the literature to find the best suitable candidate reference gene for the goat. We observed that in almost infield studies, ACTB has been used as the choice reference gene in several similar studies in the goat (19), bovine (3, 9, 20). Moreover, ACTB was selected as a suitable internal control for study of gene expression in cryopreserved egg and embryo because it efficiently withstands cryoshocks and oocyte manipulation (9, 17, 21). After ascertaining that the expression of ACTB was stable among different development stages of embryos (data not shown), relative quantification of the target genes was undertaken with ACTB as the reference gene. For RT-qPCR, total RNAs of oocytes and embryos were extracted and used for cDNA synthesis. RT-qPCR was carried out using 1 µl of cDNA (50 ng), 5 μl of the SYBR Green/0.2 μl ROX qPCR Master Mix (2X) (Fermentas, Germany) and 1 µl of forward and reverse primers (5 pM) adjusted to a total volume of 10 µl using water nuclease-free. Three technical replicates of RT-qPCR were conducted for each primer. CT samples of each target gene were normalized to the CT of ACTB and represented as 2^-ΔΔCT^ (22). The primer sequences, annealing temperatures and the size of amplified products are shown in Table 1.

**Table 1:** Specific real-time primers were designed for gene sequences


Gene	Primer sequences (5ˊ-3ˊ)	Length of PCR product	TM

*Lifr1*	F: ATTTTTCGGTGTATGGGTGC	117	56
	R: CAGATGTATCCTCAACGGTA		
*Bmpr1*	F: CCTGTTCGTCGTGTCTCAT	116	58
	R: CTGGTGCTAAGGTTACTCC		
*Alk4*	F: TCTCCAAGGACAAGACGCTC	152	62
	R: ACGCCACACTTCTCCAAACC		
*Smad1*	F: TCACCATTCCTCGCTCCCT	140	60
	R: AAACTCGCAGCATTCCAACG		
*Smad5*	F: ACAGCACAGCCTTCTGGTTC	136	60
	R: GGGGTAGGGACTATTTGGAG		
*Id3*	F: CGGCTGAGGGAACTGGTA	198	58
	R: CCTTTGGTCGTTGGAGATG		
*Ctnnb*	F: AGTGGGTGGCATAGAGG	160	54
	R: CACAGGTAGCCCGTAG		
*Akt*	F: TTCAGCAGCATCGTGTGGCA	98	60
	R: TCATCAAAATACCTGGTGTCCG		
*Oct4*	F: GCCAGAAGGGCAAACGAT	96	56
	R: GAGGAAAGGATACGGGTC		
*Rex1*	F: GCAGCGAGCCCTACACAC	94	61
	R: ACAACAGCGTCATCGTCCG		
*Fzd*	F: CATCGGCACTTCCTTTATCC	89	59
	R: GCTTGTCCGTGTTCTCCC		
*c-Myc *	F: CAACACCCGAGCGACACC	160	61
	R: GCCCGTATTTCCACTATCCG		
*Sox2*	F: ATGGGCTCGGTGGTGA	182	54
	R: CTCTGGTAGTGCTGGGA		
*Fgfr4*	F: GCTGACTGGTAGGAAAGG	193	56
	R: AGTGGCTGAAGCACATCG		
*Nanog*	F: GATTCTTCCACAAGCCCT	137	54
	R: TCATTGAGCACACACAGC		
*Erk1*	F: TCAAGCCGTCCAACATCCT	204	58
	R: CGACCGCCATCTCAACC		
*Gata4*	F: TCCCCTTCGGGCTCAGTGC	128	64
	R: GTTGCCAGGTAGCGAGTTTGC		
*Cdx2*	F: CCCCAAGTGAAAACCAG	144	53
	R: TGAGAGCCCCAGTGTG		
*Cdc25a*	F: TGGCAAGCGTGTTATCGTG	119	58
	R: GGTAGTGGAGTTTGGGGTA		
*Actb*	F: CCATCGGCAATGAGCGGT	146	60
	R: CGTGTTGGCGTAGAGGTC		

PCR; Polymerase chain reaction and TM; Melting temperature.

#### Statistical analysis

Statistical analysis were carried out using SPSS software. For the analysis of developmental data and real-time PCR data a two-tailed Students t test with equal variance was used to determine significance data. Statistical significance was accepted at P<0.05.

### Results

All the 19 genes examined were expressed throughout embryo development, from MII-oocyte to D14 developing blastocysts (23, 24). Even though, the levels of expression of all genes varied during different developmental stages as no gene was found to be stably expressed throughout embryo development. Moreover, different genes had different levels of expression with respect to a certain stage of development. Stage profiling revealed robust and dynamic patterns of stage-specific gene activity that fall into four major clusters depending on the respective mRNA profile (Figes.2-6). 

**Fig.2:**
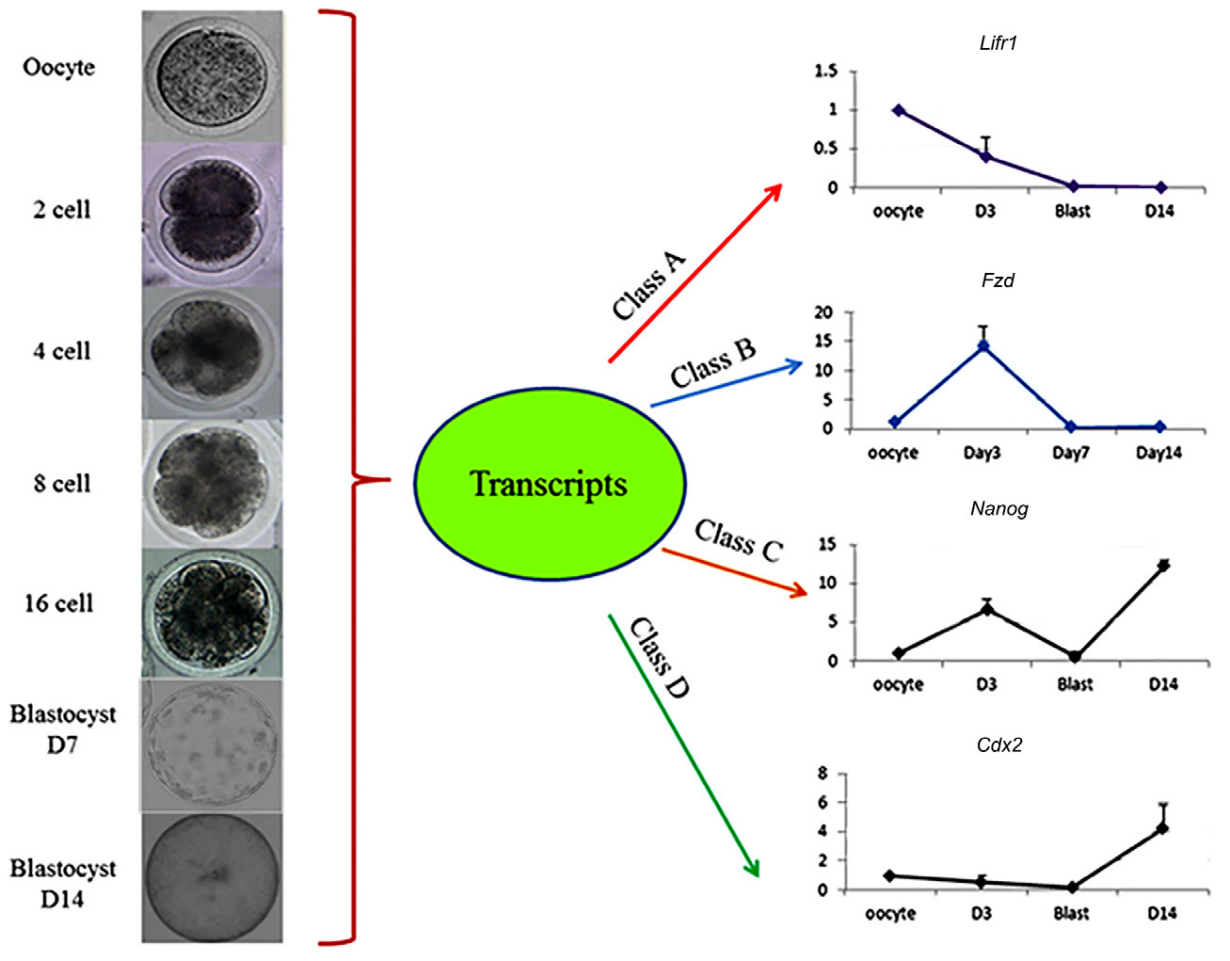
Schematic design for classification of profiles of gene expressions.

The first gene cluster (Figes.2, 3) exhibited highest levels of mRNA in MII-oocyte which gradually and consistently decreased during subsequent stages of embryo development. This cluster encompassed 11 genes including *Lifr1, Bmpr1, Alk4, Smad1, Smad5, Id3, Ctnnb, Akt, Oct4, Erk1* and *Rex1*. The speed and extent of the stepwise decreases in the transcripts varied between the genes. 

**Fig.3:**
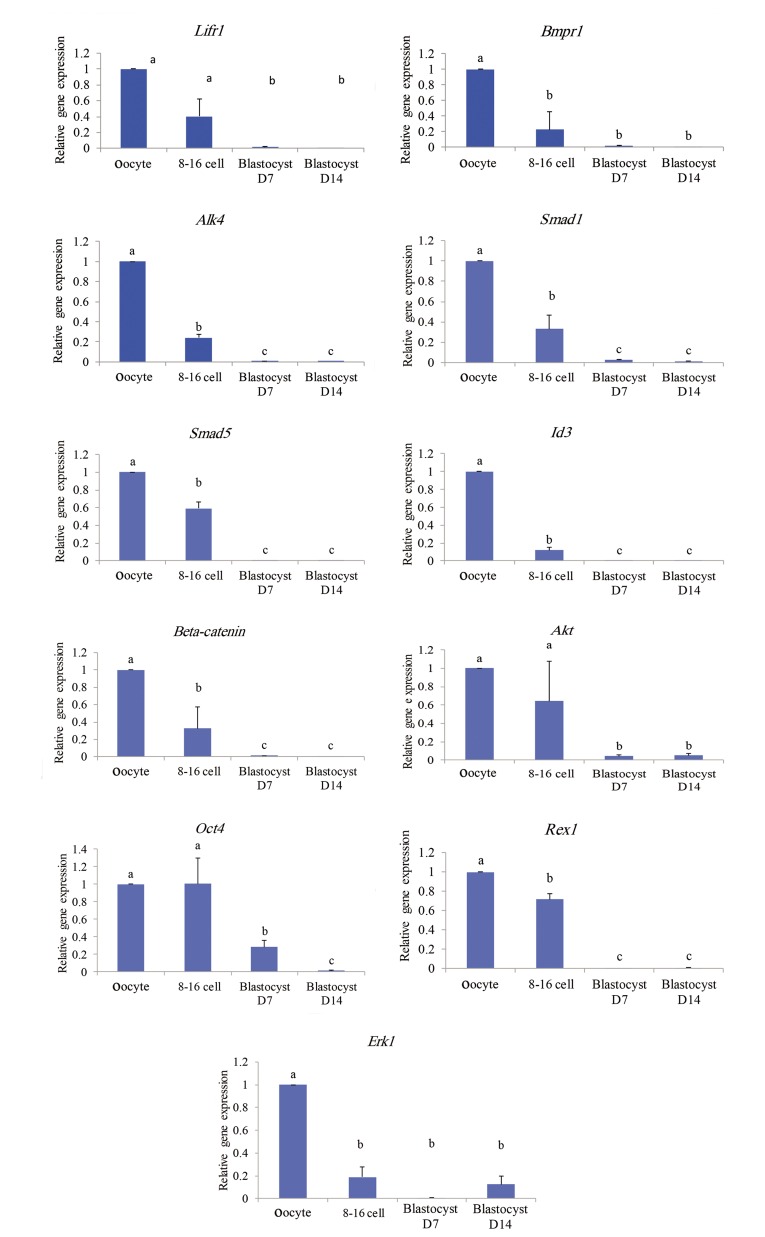
Profiles of expression of genes categorized in the first class based on Figure 2. a, b, and c; Significant difference at P<0.05%.

The second gene cluster (Figes. 2, 4) showed low levels of the transcripts in MII-oocytes, reached their highest relative mRNA levels in D3 embryos and significantly decreased thereafter with no sign of regain in transcription in D7 and D14 blastocysts. This gene set encompassed 4 genes including *Fzd, Sox2, c-Myc,* and *Cdc25a* which showed 14-, 22-, 60-, and 3- fold increase in their D3 transcripts compared to MII-oocytes. 

**Fig.4:**
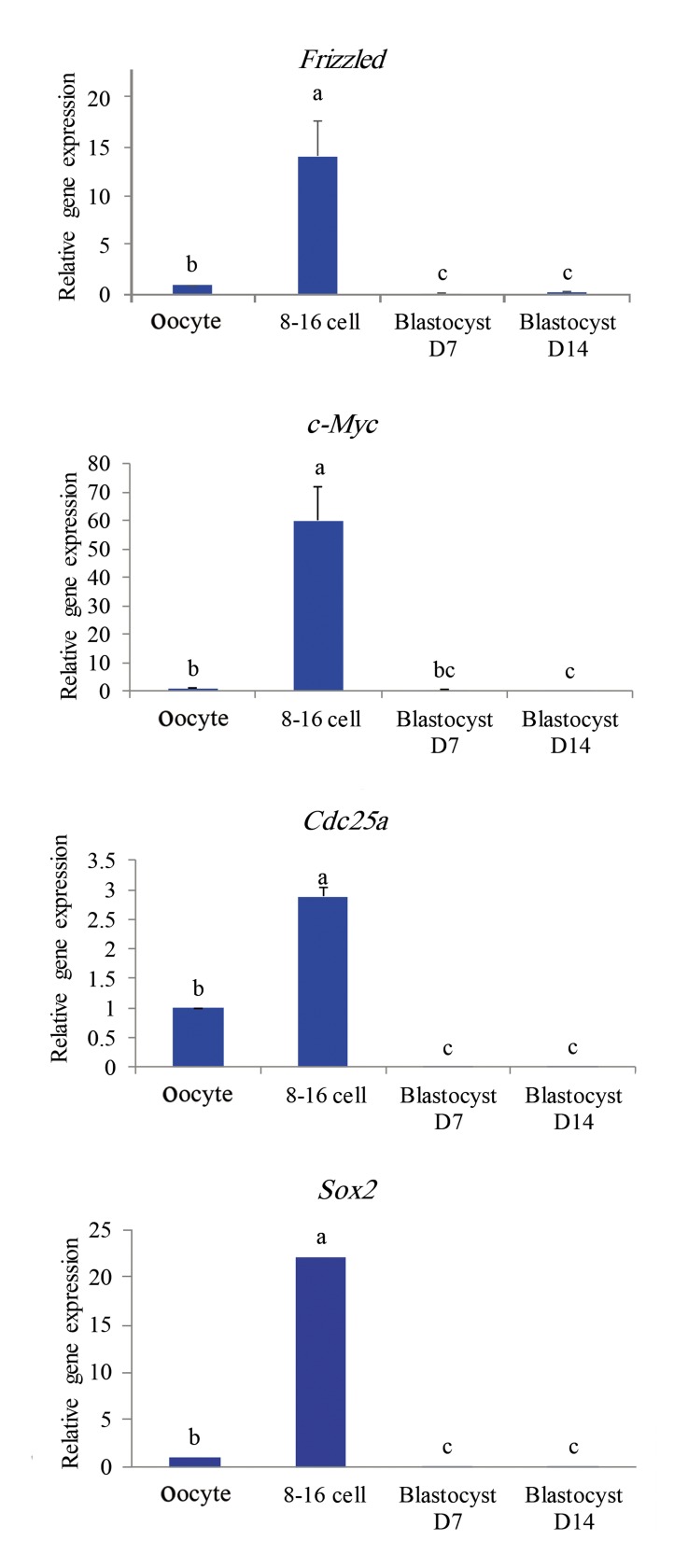
Profiles of expression of genes categorized in the second class based on Figure 2. a, b, and c; Significant difference at P<0.05%.

In the third gene cluster (Figes.2, 5), the original levels of transcripts of MII-oocyte gradually decreased in developing embryos with a significant reduction in D7 blastocysts. However, these genes initiated transcription from D7 onwards which resulted in a peak of expression in D14 blastocysts. This gene set composed of 2 genes, *Gata4* and *Cdx2*. Despite similar pattern of expression, the magnitude of D14 gain in transcription was different between *Gata4* and *Cdx2* (4- and 2-fold, respectively) compared to the relative mRNA levels initially found in MII-oocytes.

**Fig.5:**
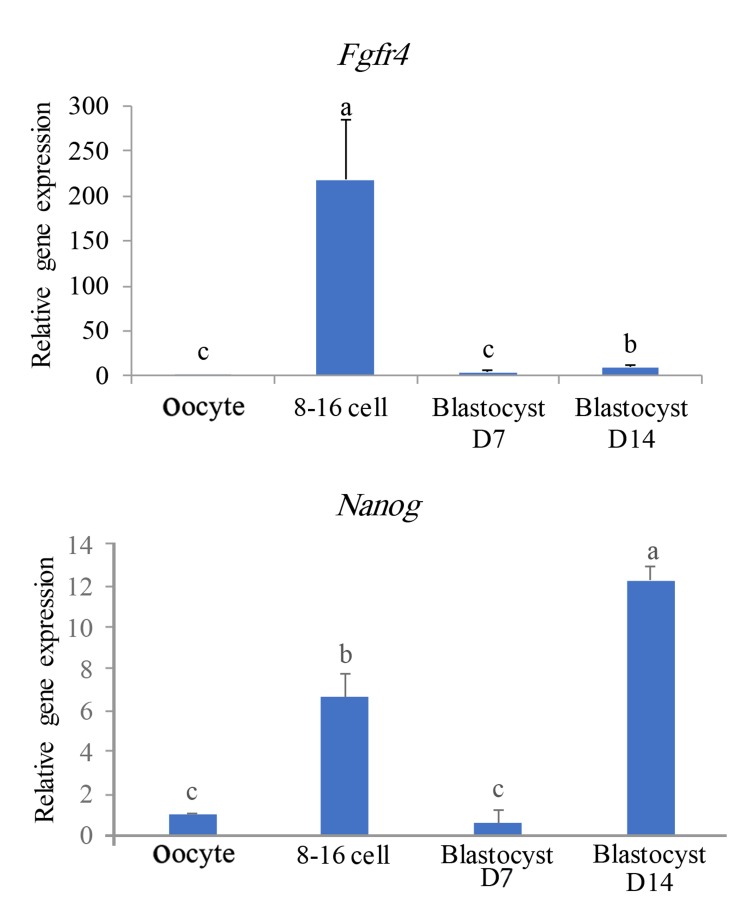
Profiles of expression of genes categorized in the third class based on Figure 2. a, b, and c; Significant difference at P<0.05%.

The fourth gene cluster (Figes.2, 6), showed low levels of the transcripts in MII-oocytes, reached their first the peak of expression in D3 embryos and followed by a significant decrease in transcription in D7 blastocysts. However, this group of genes reinitiated transcription from D7 onwards which resulted in the second peak of expression in D14 blastocysts. This gene set encompassed 2 genes including *Fgfr4* and *Nanog*. 

**Fig.6:**
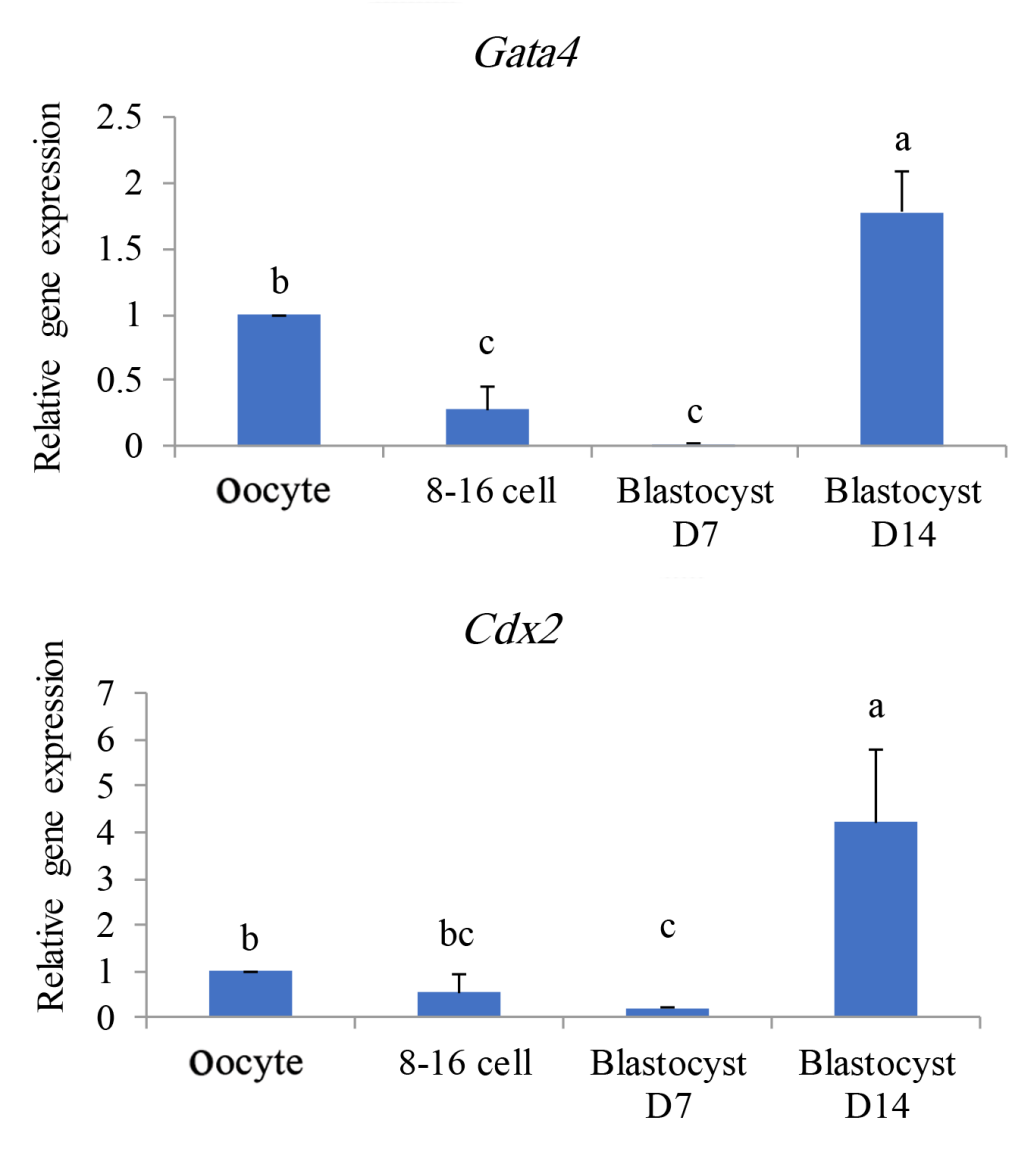
Profiles of expression of genes categorized in the fourth class based on Figure 2. a, b, and c; Significant difference at P<0.05%.

## Discussion

This study demonstrated that all developmental genes assessed are present throughout the pre and peri implantation stages of goat *in vitro* embryo development. Even though, none of these genes could exhibit significantly stable and ubiquitous expression patterns throughout these five developmental stages. Instead, all genes showed fluctuations in expression levels, and if we exclude *Oct4* and *Rex1*, the source of the greatest variations in relative mRNA expression was between MII-oocyte and D3 stages of embryo development. To better explain the quantitative results, schematic patterns of gene expression were categorized in the context of four groups based on the actual patterns of gene expression observed.

The first set of genes, which importantly comprised two-third of the examined genes, revealed a consistent trend of gradual mRNA reduction as the highest and lowest levels of transcripts were observed in MII-oocyte and D14 blastocysts, respectively. This may suggest that the transcripts of these set of genes have been transcribed and accumulated during oocyte growth phase. Because MII-oocyte and early embryo are considered transcriptionally silent (25) and since there is no evidence of active transcription during meiosis resumption, these maternal transcripts should be produced during earlier stages of oocyte growth preceding germinal vesicle breakdown stage. 

Theoretically, the steady state of mRNA reduction without renewal (gene cluster 1) may suggest the potential importance of these mRNA for production of proteins that are required during early stages of embryo development, especially to support maternal embryonic transition (MET), when the goat embryos are self-reliant in their transcription. Mechanistically, the distinctive processes have been associated with the declines of maternal mRNA in the eukaryotes including deadenylation, degradation, and protein translation or synthesis (26). Moreover, it has been suggested that the oocyte unlikely would keep useless products (27). Therefore, the quick reduction in relative mRNA abundance could be associated with the protein production.

The second group of genes revealed a significant elevation in their transcripts at D3 compared to MII-oocyte. But, this elevation in the transcripts did not continue and gradually declined and reached the lowest levels in D14 blastocysts . The potential importance of D3 burst-in-the-transcription is indicative of their crucial importance at the MET stage. Accordingly, the lower abundances of these transcripts during post-MET period may not play an important role in the regulation of blastocyst formation and further stages.

The third group of genes revealed a gradual decrease in the maternal mRNA abundances similar to the first group, but their transcripts increased from D7 and reached their highest levels at D14. This would imply that this set of genes is of critical importance during pre- and post- MET phases and underscores the facts that: i. The exact time windows that the second and third sets of genes are in demand for the embryo development are different, and ii. The maternal stockpiles of the third, but not second, set of genes are quite enough to support MET without any need for the additional source of embryo-specific transcripts. 

The fourth group of genes was those showing two peaks in embryo-specific mRNA transcription at two distinctive time points of MET and day 14 blastocyts. This may suggest that these transcripts are crucially needed during both stages. This group can be considered as the combined model of the second and third groups, and correspondingly, the genes in this final group may be of theoretical alternative capacity to cover the duties of genes in both second and third groups.

One of the shortcomings of this study was usage of *in vitro* derived embryos while *in vivo* -derived embryos provide the best source of samples for gene expression studies. However, this was not possible due to technical limitations. A wide range of infield studies have used *in vitro* -derived embryos for similar analyses in the goat (9), equine (28), and bovine (3, 20). In ungulates, embryos are migrating within the uterus for about 7 days before implantation (12). This delay in implantation has provided a unique opportunity to extended in vitro culture of ungulates (28-30). At this stage, we expected to see day 14 blastocysts but a search in filed studies revealed that at this time window, embryos look similar to the blastocysts observed on day 14 in this study. For better clarifying this issue and also confirm the quality of these embryos to continue to development, we had previously transferred a number of *in vitro* derived day-7 blasto- cysts into uterine horn of synchronized goat recipients as routine. The transferred embryos were then flushed from the uteruses at day 20-21. Surprisingly enough, we observed that the embryos at day 21 start to form ovoid or tubular like structures with sizes ranged 0.5 to 5 mm in diameter. One may suggest that the elongation process of goat embryos possibley begins on day 14 onwards. 

## Conclusion

The results obtained through this work highlight the fact that transcription factors involved in the regulation of early events of pluripotency and differentiation are present through pre- and periimplantation in the goat embryos. Since the blastocysts of ungulates, unlike human and mice, implant with a delay of around 7 days, the obtained results in D7 and 14 blastocysts may provide useful information to figure out the expression profiles of developmentally important genes between these two stages. The profiles obtained may be useful for derivation of ESC in this valuable farm species. 
